# The role of nutrition and multimodal lifestyle interventions in Alzheimer’s prevention and management: a mini-review

**DOI:** 10.3389/fnut.2026.1818913

**Published:** 2026-04-08

**Authors:** Hailong Zhang, Chenyang Liu, Xingxing Yuan, Hongna Yin

**Affiliations:** 1 Department of Acupuncture, Second Affiliated Hospital of Heilongjiang University of Chinese Medicine, Harbin, Heilongjiang, China; 2 First Clinical Medical College, Heilongjiang University of Chinese Medicine, Harbin, China; 3 Department of Medicine, Heilongjiang Academy of Traditional Chinese Medicine, Harbin, Heilongjiang, China

**Keywords:** Alzheimer’s disease prevention, dietary interventions, lifestyle intervention, multidomain, personalized nutrition

## Abstract

Alzheimer’s disease (AD) is a progressive and currently incurable neurodegenerative disorder, which is driving a paradigm shift in research focus toward preventive and disease-modifying strategies. This mini-review synthesizes current evidence on dietary and lifestyle interventions for AD prevention and management from randomized controlled trials (RCTs) and observational studies. Current findings indicate that multidomain approaches, such as the Mediterranean-DASH Intervention for Neurodegenerative Delay (MIND) diet and the Finnish Geriatric Intervention Study (FINGER) model, which integrate nutrition, physical activity, and cognitive training, consistently demonstrate efficacy in slowing cognitive decline and reducing brain atrophy in at-risk elderly populations. The evidence for specific nutritional supplements is mixed; however, certain combinations like omega-3 fatty acids paired with carotenoids, B vitamins (folate/B12), and probiotics show promise, particularly for improving memory and reducing inflammation. Intervention outcomes are significantly influenced by genetic factors, especially the APOE4 carrier status, which modulates nutrient metabolism and amyloid response, thereby underscoring the critical need for personalized approaches. Key targeted biological pathways include oxidative stress, phospholipid metabolism, and neuro-inflammation. Despite promising data, several limitations persist, such as inconsistent results, short trial durations, and a lack of standardized protocols. Future research must prioritize long-term, genetically stratified trials alongside mechanistic studies to validate efficacy, optimize personalization, and translate findings into clinically actionable, scalable guidelines for diverse populations.

## 1 Introduction

Alzheimer’s disease (AD) is a progressive neurodegenerative disorder for which no cure currently exists, thereby shifting the research emphasis toward preventive and disease-slowing strategies (1, 2). Numerous randomized controlled trials (RCTs) and observational studies have explored a range of dietary and lifestyle interventions aimed at preventing or slowing cognitive decline. Among these, multidomain approaches have demonstrated particular promise. For instance, a three-year RCT evaluating the Mediterranean-DASH Intervention for Neurodegenerative Delay (MIND) diet showed its potential to slow cognitive decline and reduce brain atrophy in at-risk older adults, as assessed by a validated cognitive battery (3–5). Similarly, the two-year Finnish Geriatric Intervention Study (FINGER), a multidomain program encompassing diet, exercise, and cognitive training, was shown to improve global cognition in at-risk elderly, with enhanced benefits observed in carriers of the apolipoprotein E ε4 (APOE4) allele (6–8). Subsequent analyses have further revealed that genetic risk profiles, including APOE4 status, can influence responses to interventions targeting factors such as amyloid deposition, thereby underscoring the need for personalized strategies (9, 10).

The evidence for specific nutritional supplements, however, is mixed. Combinations such as omega-3 fatty acids with carotenoids and vitamin E have been associated with improvements in working memory, while probiotics like *Bifidobacterium longum* and the microalgae spirulina have shown potential in reducing inflammation and enhancing cognition (11–13). Conversely, other supplements, including inosine for Parkinson’s disease and low-dose leuco-methylthioninium bis (hydromethanesulfonate) (LMTM) for AD, failed to demonstrate cognitive benefits (14, 15). Synergistic effects have been observed between folate and vitamin B12 in improving cognition and reducing homocysteine levels in individuals with mild cognitive impairment (MCI) (16, 17). Additionally, compounds like genistein and betaine have shown promise in the prodromal AD stage by modulating amyloid and metabolic markers (18, 19).

Multimodal interventions that integrate dietary guidance, physical activity, and cognitive stimulation have consistently yielded benefits. The MIND-ADmini pilot trial reported improved dietary quality and cognitive stability in patients with prodromal AD (20–22). Furthermore, resistance exercise has been shown to preserve hippocampal structure in MCI, and personalized lifestyle interventions have led to improved cognitive scores (23, 24). Large-scale international efforts, such as the United States Study to Protect Brain Health Through Lifestyle Intervention to Reduce Risk (U.S. POINTER), are now adapting successful models like FINGER to assess the efficacy of multidomain strategies in more diverse populations (25).

Emerging genetic and biomarker insights are continuously refining our understanding of these interventions. In particularly, the APOE4 genotype has been shown to modulate nutrient metabolism and amyloid pathology, suggesting that critical nutrient-gene interactions may underlie the differential responses observed in clinical studies (26). While specific nutrient combinations, such as Fortasyn Connect, have shown modest cognitive benefits in prodromal AD, other trials, including the Multidomain Alzheimer Preventive Trial (MAPT), found that omega-3 supplementation combined with a multidomain intervention did not significantly slow cognitive decline (27, 28). Interestingly, some interventions, like virgin coconut oil, showed no overall benefit but may aid specific subgroups such as APOE4 carriers (29). Cultural adaptations, as exemplified by studies on matcha tea improving emotional perception and sleep in individuals with mild cognitive decline, are also being explored (30).

In summary, current evidence supports the value of multidomain lifestyle interventions and targeted nutritional strategies, such as the MIND diet and omega-3 supplementation, in slowing cognitive decline. Personalizing approaches based on genetic risk and baseline biomarker profiles holds promise for optimizing outcomes, though longer-term RCTs are needed to confirm sustained efficacy and elucidate underlying mechanisms. This mini-review aims to critically synthesize the current evidence from RCTs and observational studies on the efficacy of dietary and lifestyle interventions for preventing and managing AD. Specifically, it seeks to analyze the effectiveness of these specific nutritional strategies, including the MIND diet, supplements, and multimodal approaches, in slowing cognitive decline. Furthermore, it will elucidate the biological pathways involved, examine how genetic heterogeneity, particularly APOE4 carrier status, influences intervention outcomes, and discuss limitations of existing research to inform the development of future personalized prevention strategies and clinical guidelines.

## 2 AD prevention through dietary supplements

Emerging evidence indicates that dietary supplements may play a role in AD prevention, although their efficacy varies considerably across interventions. To provide clarity, this evidence is organized below by supplement type.

### 2.1 Fatty acid supplementation

The effects of omega-3 fatty acids (DHA/EPA) have been mixed and appear highly context-dependent. One study demonstrated that a combination of omega-3 fatty acids with other nutrients; specifically, carotenoids such as lutein and meso-zeaxanthin, and vitamin E, led to improvements in working memory among older adults (11, 31). In contrast, supplementation with omega-3 fatty acids alone showed no overall cognitive benefit in patients diagnosed with AD, although modest improvements were observed in a subgroup of those who were APOE4-negative (32). Multidomain trials that combined omega-3 supplementation with lifestyle interventions such as diet and exercise have yielded mixed outcomes, with no significant slowing of cognitive decline reported in the primary analyses (33).

### 2.2 Vitamin supplementation

Folate and vitamin B12 supplementation have been shown to reduce homocysteine levels and improve cognitive performance in individuals with MCI, with combined administration suggesting synergistic benefits (34). Long-term *β*-carotene supplementation, as observed in the Physicians’ Health Study, was associated with attenuated cognitive decline, particularly in verbal memory. However, safety considerations remain important; high-dose vitamin E supplementation at 2000 Units per day may require monitoring for potential adverse effects, including bleeding risk (35).

#### 2.2.1 Probiotic supplementation

Probiotic interventions have shown potential in modulating inflammation and cognition. The probiotic *B. longum* BB68S was found to enhance cognitive function in healthy older adults, an effect that correlated with an increase in beneficial gut bacteria (36). Other probiotic formulations have also been shown to reduce inflammatory markers such as high-sensitivity C-reactive protein and oxidative stress in patients with mild-to-moderate AD (37, 38).

In a 12-week trial, spirulina supplementation was associated with improved Mini-Mental State Examination (MMSE) scores and reduced insulin resistance in AD patients (35, 39). While these preliminary findings are encouraging, it is important to interpret them with caution. The short intervention period is insufficient to evaluate meaningful long-term cognitive change, and the proposed anti-inflammatory and antioxidant mechanisms, though plausible, remain speculative and are not directly proven by the reported outcomes.

#### 2.2.2 Emerging nutraceutical compounds

Several other compounds have shown promise in early-phase trials. In individuals with prodromal AD, genistein, a soy isoflavone, improved verbal memory and stabilized amyloid-β deposition in the anterior cingulate cortex, indicating potential disease-modifying effects (18). Daily consumption of matcha tea was associated with improved emotional perception and sleep quality in older adults with cognitive decline, although primary cognitive endpoints remained unchanged (30, 40). The efficacy of these supplements is often influenced by genetic factors such as APOE4 status and baseline biomarker profiles.

**Figure 1:**
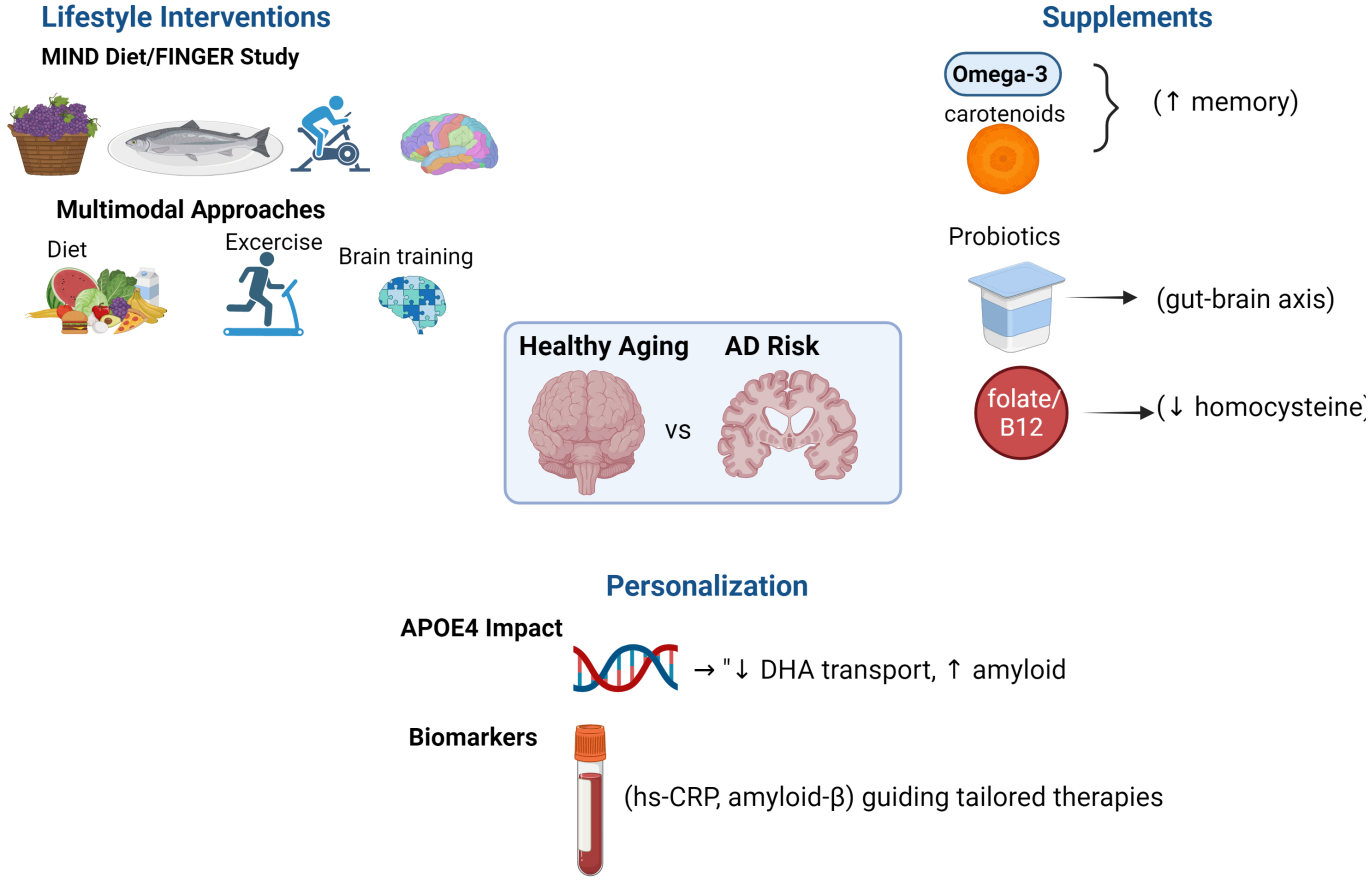
Evidence-based medicine in Alzheimer’s disease prevention and management.

Notably, dietary supplements appear to yield greater benefits when integrated into broader dietary patterns. For instance, the MIND diet, which incorporates several nutrient-rich components, has been shown to slow cognitive decline and reduce brain atrophy in at-risk populations (3–5). In summary, while mechanistic support for many supplements is compelling, clinical outcomes have been inconsistent. Further large-scale, long-term trials are necessary to validate these findings and establish optimized dosing and personalization strategies Figure 1.

## 3 Dietary interventions and biological pathways in AD and cognitive decline

Emerging research underscores the potential of dietary interventions to modulate key biological pathways implicated in AD progression, including oxidative stress, phospholipid metabolism, neuro-inflammation, and metabolic regulation (41). Clinical trials have reported cognitive improvements through the use of nutritional supplements, medical foods, and regimens involving caloric restriction. However, while changes in metabolic or oxidative stress markers are frequently observed, their direct relevance to cognitive outcomes and AD pathophysiology requires further validation. Personalized approaches that account for genetic and metabolic variability may enhance intervention efficacy, though large-scale validation remains necessary to establish robust clinical guidelines. The evidence is organized below according to the primary biological pathway or intervention type.

### 3.1 Antioxidant-related interventions

Several interventions appear to exert effects through modulation of oxidative stress. A four-month supplementation with fenugreek seed extract significantly improved memory and oxidative stress markers in individuals with mild-to-moderate AD, as reflected by increased total antioxidant capacity and reduced malondialdehyde levels (42, 43). Similarly, flavanol-rich cocoa improved memory function in older adults with suboptimal nutrition, especially in hippocampal-dependent tasks, an effect potentially linked to improved vascular function and antioxidant activity (44, 45). Walnut consumption has also been associated with enhanced cognitive performance in healthy older adults, though no specific benefits were observed in AD cohorts, and the precise antioxidant mechanisms in humans remain to be fully elucidated (46).

### 3.2 Lipid metabolism and membrane-related compounds

Compounds targeting phospholipid metabolism and neuronal membrane integrity have shown promise, particularly in early disease stages. Scallop-derived plasmalogens administered to individuals with mild AD or MCI showed memory improvements in female participants and those aged under 77 years, despite no overall cognitive benefit, suggesting a potential role in membrane restoration (47). Fortasyn Connect, a multi-nutrient blend containing DHA, EPA, uridine, choline, and vitamins, slowed cognitive decline and reduced hippocampal atrophy in prodromal AD patients over 24 months, with effects attributed to enhanced synaptic formation and membrane phospholipid synthesis (27, 48). Its medical food counterpart, Souvenaid, improved phospholipid metabolism and nutritional biomarkers in mild AD patients (49). Additionally, whey protein enriched with milk fat globule membrane (MFGM), taurine, and B vitamins enhanced memory and processing speed in individuals with MCI after 12 months, likely through combined effects on neuronal membrane composition and homocysteine metabolism (50, 51).

The effects of omega-3 fatty acid supplementation further illustrate the complexity of targeting lipid pathways. While omega-3 supplementation was found to increase neuro-filament light chain; a biomarker of axonal injury and neurodegeneration in individuals with AD, without altering amyloid-*β* levels, its cognitive benefits appear to be strongly influenced by genetic background (11). Specifically, omega-3 supplementation improved cognitive outcomes in APOE4-negative individuals, whereas APOE4 carriers exhibited impaired transport of docosahexaenoic acid to the cerebrospinal fluid (32, 52).

### 3.3 Food-based and multinutrient interventions

Broader dietary patterns and multinutrient interventions may simultaneously engage multiple pathways. Supranutritional supplementation with sodium selenate elevated central nervous system selenium levels, suggesting potential benefits in AD through antioxidant and anti-protein aggregation mechanisms, though direct cognitive evidence remains preliminary (44). Long-term β-carotene supplementation, as observed in the Physicians’ Health Study, was associated with attenuated cognitive decline, particularly in verbal memory, potentially via sustained antioxidant protection (43). Intermittent caloric restriction, studied in models such as multiple sclerosis with implications for AD, was associated with increased cortical volume and reduced neuro-inflammatory markers, suggesting metabolic and anti-inflammatory effects (53). Conversely, high-glycemic diets have been linked to elevated cerebral amyloid burden in cognitively normal older adults, underscoring the importance of overall glycemic control in metabolic pathways relevant to AD (54).

### 3.4 Anti-neuroinflammatory interventions

**Figure 2:**
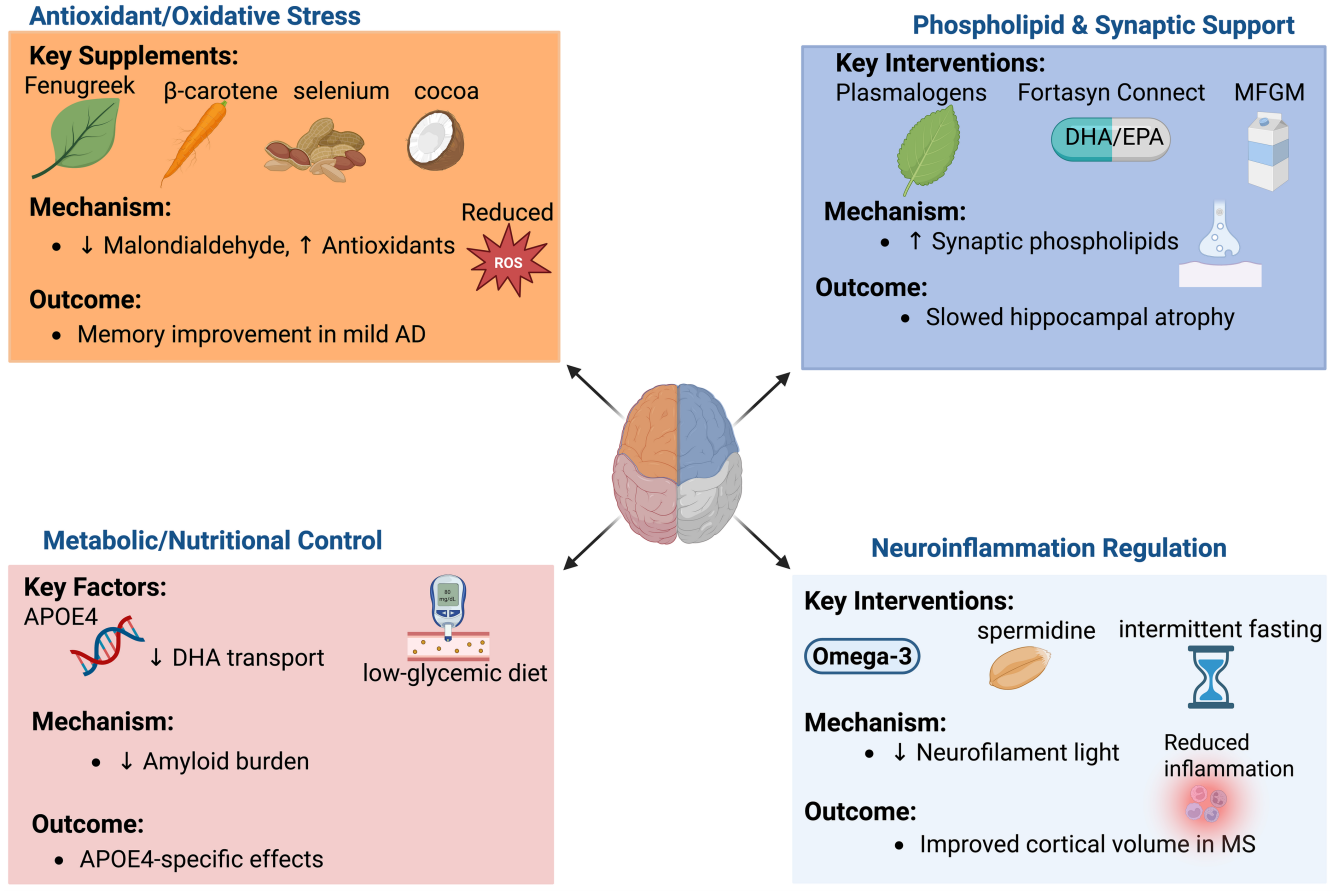
Dietary interventions and biological pathways in AD and cognitive decline.

Targeting neuro-inflammation represents another promising avenue. Spermidine supplementation has demonstrated efficacy in improving memory and mood in older adults with subjective cognitive decline, potentially through autophagy induction and anti-neuroinflammatory mechanisms (52). Probiotic formulations, including *B. longum*, have been shown to reduce systemic inflammatory markers, though direct evidence of central neuro-inflammation modulation in humans remains limited (36–38) Figure 2.

## 4 Limitations

This review highlights several important limitations in current research on dietary interventions for AD. First, the evidence remains inconsistent for many interventions: while some trials report cognitive benefits with Fortasyn Connect or fenugreek seed extract, others show no significant effects for the MIND diet and virgin coconut oil, raising concerns regarding reproducibility and the influence of contextual factors. Second, genetic and metabolic heterogeneity significantly affects outcomes. For instance, APOE4 carriers exhibit impaired DHA transport in response to omega-3 supplementation, and medium-chain triglycerides show benefit only in non-carriers, underscoring the need for personalized approaches that are not yet fully developed (11). Third, the scarcity of long-term data limits the validity of conclusions; many trials, including those investigating Fortasyn Connect and spermidine, span 24 months or less, which is insufficient to evaluate sustained efficacy or disease-modifying potential.

From a mechanistic standpoint, pathways linking nutritional interventions to AD biomarkers such as amyloid-*β* and tau remain poorly validated. For example, while folic acid and probiotics can reduce inflammation, direct evidence of amyloid modulation is lacking. Similarly, biomarker of axonal injury and neurodegeneration markers such as neuro-filament light chain correlate inconsistently with clinical outcomes (55). A lack of standardization also impedes progress; studies vary widely in cognitive assessments tool, metrics of dietary adherence, and biomarker thresholds, complicating cross-trial comparisons. Practical barriers, including socioeconomic disparities in access to specialized diets such as plant-based protocols, along with adherence challenges in advanced AD due to dysphagia or behavioral symptoms, remain understudied. The timing of interventions is another unresolved issue; most trials focus on prodromal or mild AD, leaving preventive strategies for preclinical stages largely unexplored. Finally, safety concerns accompany high-dose supplements such as vitamin E and omega-3 fatty acids, necessitating careful monitoring for adverse effects including bleeding risk (40). Although multimodal approaches combining diet, exercise, and cognitive training show promise, their scalability and cost-effectiveness have not been adequately evaluated. Together, these limitations emphasize the need for larger, longer-term RCTs that adopt stratified designs based on genetics and baseline biomarkers, implement standardized protocols, and incorporate real-world feasibility assessments to translate dietary strategies into clinical practice.

## 5 Future directions

Future research should prioritize long-term, large-scale RCTs to validate the sustained efficacy of dietary interventions in AD. Extending trials such as those involving Fortasyn Connect or fenugreek seed extract beyond 24 to 36 months could clarify their disease-modifying potential and safety in diverse populations (56). Stratified trial designs that enroll participants based on APOE4 status, baseline amyloid-β and tau biomarkers, and gut microbiota profiles are essential to identify subgroups most likely to benefit from specific interventions (57, 58). Mechanistic studies should elucidate nutrient-biomarker interactions, such as how selenium or spermidine influences neuro-inflammation or whether probiotics modulate amyloid clearance via the gut-brain axis (59, 60).

Standardized protocols for cognitive assessments, dietary adherence monitoring, and biomarker measurement are urgently needed. Harmonizing thresholds for oxidative stress markers including malondialdehyde, and neuro-inflammatory indices such as neuro-filament light, would significantly improve cross-trial comparability. Research should also explore earlier intervention windows, targeting preclinical AD or high-risk groups such as APOE4 carriers to evaluate preventive potential. Trials integrating nutrition with lifestyle components such as exercise and cognitive training must assess scalability and cost-effectiveness, particularly in low-resource settings (61). Real-world feasibility studies are necessary to address access barriers and adapt interventions, such as matcha tea or region-specific probiotics, to diverse cultural contexts. By integrating omics technologies such as genomics and metabolomics with clinical outcomes, future studies can advance personalized nutrition frameworks and translate preclinical insights into clinically actionable guidelines.

## 6 Conclusion

Based on the extensive evidence reviewed, dietary and lifestyle interventions demonstrate significant, though complex, potential in the prevention and management of AD. The most consistent support exists for multimodal strategies, such as those employed in the FINGER and MIND trials, which integrate dietary patterns, physical activity, and cognitive training. These approaches have been shown to slow cognitive decline and reduce brain atrophy in at-risk populations. Targeted nutritional supplementation, including omega-3 fatty acids, B vitamins for homocysteine reduction, and multi-nutrient formulations such as Fortasyn Connect, also shows efficacy, particularly in prodromal and mild AD stage.

However, results are not universally positive, and considerable heterogeneity in treatment response underscores the impact of individual factors such as genetic predisposition, biomarker profiles, and metabolic characteristics. This variability highlights the imperative for personalized nutritional approaches. Translation these findings into clinical practice remains challenging due to limitations such as insufficient long-term data, inconsistent outcomes across studies, and a lack of standardization in trial design and assessment methods. Future work should focus on long-term, stratified randomized controlled trials that account for genetic and biomarker variability, along with standardized protocols and real-world feasibility studies. Ultimately, advancing from generalized recommendations toward personalized, precision nutrition frameworks, combined with multimodal lifestyle strategies, offers the most promising path to developing effective, scalable, and culturally adaptable approaches to mitigate the global burden of AD.
